# Antioxidative Effects of Natural Products on Diabetic Cardiomyopathy

**DOI:** 10.1155/2017/2070178

**Published:** 2017-10-18

**Authors:** Bingdi Yan, Jin Ren, Qinghua Zhang, Rong Gao, Fenglian Zhao, Junduo Wu, Junling Yang

**Affiliations:** ^1^Department of Respiratory Medicine, The Second Hospital of Jilin University, 218 Ziqiang Street, Changchun 130041, China; ^2^Department of Clinical Laboratory, The Second Hospital of Jilin University, 218 Ziqiang Street, Changchun 130041, China; ^3^Department of Cardiology, The Second Hospital of Jilin University, 218 Ziqiang Street, Changchun 130041, China

## Abstract

Diabetic cardiomyopathy (DCM) is a common and severe complication of diabetes and results in high mortality. It is therefore imperative to develop novel therapeutics for the prevention or inhibition of the progression of DCM. Oxidative stress is a key mechanism by which diabetes induces DCM. Hence, targeting of oxidative stress-related processes in DCM could be a promising therapeutic strategy. To date, a number of studies have shown beneficial effects of several natural products on the attenuation of DCM via an antioxidative mechanism of action. The aim of the present review is to provide a comprehensive and concise overview of the previously reported antioxidant natural products in the inhibition of DCM progression. Clinical trials of the antioxidative natural products in the management of DCM are included. In addition, discussion and perspectives are further provided in the present review.

## 1. Introduction

Diabetes mellitus (DM) is one of the most common metabolic disorders, encountered in human populations worldwide. The number of adult diabetic patients was 285 million in 2010, and it is estimated to increase to 439 million by 2030 [1]. Persistent hyperglycemia can cause damage to various organs, including the heart, via different modes of action [2]. Amongst the numerous complications of DM, cardiovascular complications namely, hypertension, coronary heart disease, and diabetic cardiomyopathy (DCM) are the main causes of morbidity and mortality. DCM accounts for nearly 80% of the mortality noted in diabetic patients [3]. DCM is initially defined as the presence of abnormal myocardial structure and function in the absence of coronary artery disease, hypertension, and valvular disease [4]. Recent studies have proposed that DCM would be a result of a long-lasting process in which the myocardium is affected at a very early stage by metabolic changes prior to the diagnosis of DM [5]. This process progresses rapidly by the incidence of myocardial ischemia [5].

The clinical features of DCM include diastolic dysfunction at an early stage and systolic dysfunction at a late stage which result in reduced left ventricular function, early heart failure, myocardial fibrosis, and death [6]. This procedure is not accompanied by hypertension or coronary heart disease. Some patients may have no symptoms and/or mild diastolic dysfunction at the early stage, while with the progression of DCM, the patients may develop the following symptoms: shortness of breath, fatigue, weakness, and ankle edema [7].

The main cause of the pathological change of DCM is microangiopathy, which results in cardiac structural and functional alterations, such as apoptosis of the myocardium, myocardial interstitial fibrosis, and perfusion abnormality of the heart muscles. It was reported that capillary basement membrane thickening and microaneurysms were observed in patients with DCM [8, 9]. Once the myocardial interstitial fibrosis has developed, it cannot be reversed and a poor prognosis of the diseases is frequently expected. Consequently, it is imperative to identify appropriate therapeutic targets notably at the early stage of DCM.

The pathogenesis of DCM has not been fully elucidated. Various biological processes have been shown to account for the pathogenesis and progression of DCM, including, but not are limited to, oxidative stress, cardiomyocyte apoptosis, disordered calcium handling, endoplasmic reticulum stress, myocardial insulin resistance, endothelial dysfunction, mitochondrial dysfunction, and autophagy [10, 11]; amongst which, oxidative stress is believed to be a key mechanism through which DM induces DCM. Despite a few reports regarding the antioxidative effects of natural products on DCM, a systematic review, to date, has not been provided. Here, we summarize the previous findings and provide perspectives and indications for future studies.

## 2. Role of Oxidative Stress in DCM

**Figure 1:**
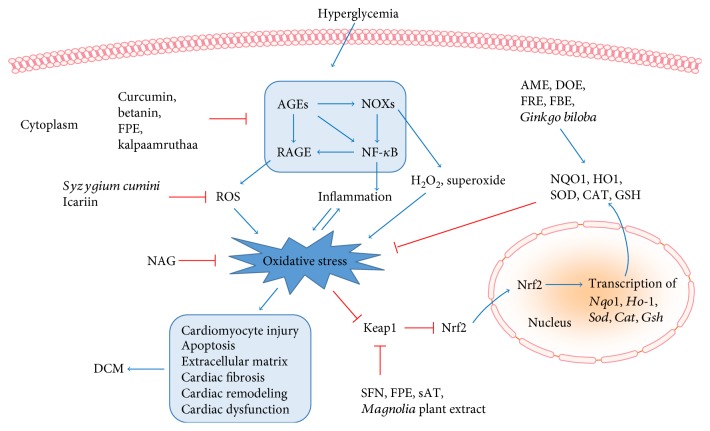
Role of antioxidative natural products in diabetic cardiomyopathy. Diabetes causes the formation of AGEs, leading to the activation of NOXs and RAGE, the effects of which induce overproduction of ROS, H2O2, and superoxide, followed by enhanced oxidative stress. AGEs can activate NF-*κ*B both directly and indirectly through NOXs, resulting in inflammation, a status that positively amplifies oxidative stress and vice versa. Consequently, the diabetes-elevated oxidative stress can cause cardiomyocyte injury, apoptosis, accumulation of extracellular matrix, cardiac fibrosis, remodeling, and dysfunction, all of which are hallmarks of DCM. These effects can be blocked or blunted by several natural products, functioning through different targets. Curcumin, betanin, FPE, and kalpaamruthaa were reported to inhibit the AGE/RAGE/NOX/NF-*κ*B pathway. *Syzygium cumini* and icariin decreased the formation of ROS. NAG had the ability to diminish diabetes-induced oxidative stress. In addition, several natural products were shown to elevate antioxidant capacity, via activating Nrf2 antioxidant system. SFN, FPE, sAT, and Magnolia plant extract inactivated Keap1, the key negative regulator of Nrf2, leading to the release of Nrf2. This effect facilitated nuclear translocation of Nrf2, resulting in the transcription of various antioxidant genes, such as *Nqo1*, *Ho-1*, *Sod*, *Cat*, and *Gsh*. As a result, these antioxidants were increased in the cytoplasm, acting as scavengers for the diabetes-induced excessive free radicals. AME, DOE, FRE, FBE, and *Ginkgo biloba* were reported to elevate the activity of these antioxidants. Collectively, the natural products, functioning either through blocking the formation of oxidative stress or through enhancing the scavenging activity, ameliorated DCM in experimental models. AGEs: advanced glycosylation end products; AME: *Aegle marmelos* leaf extract; CAT: catalase; DCM: diabetic cardiomyopathy; DOE: *Dendrobium officinale* extract; FBE: *Ficus racemosa* stem bark extract; FPE: Flos Puerariae; GSH: glutathione; HO-1: heme oxygenase-1; Keap1: Kelch-like ECH-associated protein 1; NAG: North American ginseng; NF-*κ*B: nuclear factor kappa-light-chain-enhancer of activated B cells; NOX: NADPH oxidase; NQO1: NADPH quinone oxidoreductase; Nrf2: nuclear factor erythroid 2-related factor 2; RAGE: receptor for AGEs; ROS: reactive oxygen species; sAT: *Aralia taibaiensis*; SFN: sulforaphane; SOD: superoxide dismutase; ↑: activation or improvement; ┴: inhibition or downregulation.

Reactive oxygen species (ROS) are chemically reactive chemical species containing oxygen, including peroxides, superoxide, hydroxyl radical, and singlet oxygen [12]. Mitochondrion is the main “factory” in which DM produces excessive mitochondrial superoxide [13]. The DM-induced overproduction of mitochondrial superoxide leads to increased formation of advanced glycosylation end products (AGEs), expression of the receptor for AGEs (RAGE), and activation of protein kinase C (PKC), the polyol pathway, and the hexosamine pathway [14]. In case of the excess ROS not being balanced and/or removed via the action of endogenous antioxidative enzymes and/or exogenous antioxidant molecules, an increased oxidative stress occurs, which can result in damage to proteins, lipids, and DNAs in cardiomyocytes [15]. These detrimental effects eventually lead to the remodeling of the diabetic heart, followed by its dysfunction (Figure 1).

## 3. Signaling Pathways in the Regulation of Oxidative Stress in DCM

The excess production and inefficient removal of ROS causes the induction of oxidative stress. The improvement of the antioxidative mechanisms and the suppression of the oxidative stress are considered as key targets in the treatment of DCM. Key factors, such as nuclear factor erythroid 2-related factor 2 (Nrf2), RAGE, nicotinamide adenine dinucleotide phosphate (NADPH) oxidase (NOX), and peroxisome proliferator-activated receptor (PPAR), have notably been investigated with regard to the inhibition of oxidative stress (Figure 1).

### 3.1. Nrf2 Signaling

Nrf2 is a member of the cap 'n' collar family of proteins. The gene encoding Nrf2 belongs to a subset of basic leucine-zipper (bZip) genes that was reported to act as an essential regulator of antioxidative activity and electrophilic signaling [16]. Nrf2 can promote the expression and production of detoxification enzymes and antioxidant proteins, which contribute to the clearance of ROS and the restoration of the prooxidant/antioxidant balance [17–19]. Nrf2 combines with Kelch-like ECH-associated protein 1 (Keap1), which can rapidly degrade Nrf2 through ubiquitination by proteasome [20]. Certain chemical inducers, such as heavy metals, oxidizable diphenols, and Michael acceptors, can modify the cysteine residues in Keap1 that act as nucleophiles and activate Nrf2 by suppressing the degradation of the protein [20]. Under physiological conditions, Nrf2 combines with Keap1 in the cytoplasm, whereas under oxidative stress conditions, Nrf2 dissociates from Keap1 and translocates to the nucleus. The activated Nrf2 protein then binds antioxidant-responsive elements within the promoter regions of the antioxidant genes and induces transcription of a series of antioxidant enzymes, including NADPH quinone oxidoreductase (NQO1), glutathione-S-transferase (GSH), heme oxygenase-1 (HO-1), and *γ*-glutamylcysteine synthetase [18, 21–23] (Figure 1). Nrf2 is a key protective factor in a multitude of diseases, such as cancer [24], chronic degenerative pathology [25], metal-induced toxicities [26], and angiotensin II-induced apoptosis of testicular cells [27]. Recent studies demonstrated that Nrf2 was essential in the prevention of high glucose-induced oxidative damage in cardiomyocytes, endothelial cells and vascular smooth muscle cells [28–30], and in animal models of DCM [31]. It was reported that *Nrf2* knockout mice were prone to develop severe cardiomyopathy in a streptozotocin-induced diabetic model compared with wild-type mice [31]. These findings confirmed the protective function of Nrf2 in DCM. Therefore, the activation of Nrf2 is considered as a promising therapeutic target for the treatment of DCM.

### 3.2. RAGE Signaling

RAGE is a multiligand cell surface receptor, which can be activated by a wide range of ligands, such as AGEs [32] and amphoterin [33]. RAGE is expressed in numerous normal cell types, including cardiomyocytes [34], endothelial cells [35], mononuclear phagocytic cells [36], and vascular smooth muscle cells [37]. Under diabetic condition, the DM-induced formation of AGEs binds RAGE that is expressed on the cell membrane of cardiomyocytes and endothelial cells, leading to the production of ROS, proinflammatory cytokines, and the activation of nuclear factor kappa B (NF-*κ*B). NF-*κ*B, in turn, activates the expression of RAGE [38], resulting in more severe oxidative damage. AGEs/RAGE is positively involved in the activation of NOX [39]. It has been shown that the AGE/RAGE-induced ROS interaction with NOX to generate more ROS in human endothelial cells isolated from patients with type 1 diabetes (T1DM) [39]. The data demonstrated a cross-talk between NOX and AGE/RAGE signaling, as a positive feedback loop (Figure 1). The studies proposed that the inhibition of the AGE/RAGE signaling pathway can effectively reduce DM-induced oxidative stress, thereby ameliorating DCM.

### 3.3. NOX Signaling

NOXs contribute to the production of superoxide and hydrogen peroxide (H_2_O_2_) under pathological conditions [40]. There are 7 vascular NOX isoforms in total; amongst which, NOX1, NOX2, and NOX4 are highly expressed in the diabetic heart [41]. NOX4 is the major NOX isoform that is expressed in cardiomyocytes [42] and has been demonstrated to be an important source of ROS (Figure 1). NOX4 is localized in the endoplasmic reticulum [43] and nucleus [44], interacting with NADPH as an electron donor, producing H_2_O_2_ or superoxide [45].

Increased NOX4 expression was found in the left ventricles of streptozotocin- (STZ-) induced diabetic rats [41]. This result was further confirmed in high glucose-cultured cardiomyocytes [41]. Moreover, treatment of high glucose-cultured cardiomyocytes with antisense NOX4 abrogated the high glucose-induced ROS production. In addition to the protective effect of NOX4 inhibition on high glucose- (HG-) induced cardiomyocyte injury, the beneficial effect of this approach was found in HG-treated neonatal cardiac fibroblasts as well [46], the result of which is in line with the finding that NOX4 plays an essential role in the differentiation of myofibroblasts [47, 48]. Hence, these studies suggest that the NOX4-inhibiting approach could be a promising strategy in the prevention of DCM.

## 4. Antioxidative Role of Natural Products in DCM

The antioxidative effect of natural products on the attenuation of DCM has been extensively investigated in recent years [49], showing promising outcomes. These natural products and their functions and mechanisms are listed below and in Table 1 and summarized in Figure 1.

### 4.1. Sulforaphane (SFN)

SFN, initially isolated from broccoli sprouts, is a well-known activator of Nrf2 [50] and was intensively studied for its effects in diabetic complications in recent years [18, 31, 51–54]. SFN activates Nrf2 through inactivation of Keap1, via modifying specific residues within Keap1 protein [55].

In a STZ-induced mouse model of DM, treatment with SFN for either 3 months or 6 months significantly activated Nrf2 signaling and prevented DM-induced cardiac oxidative damage, inflammation, hypertrophy, fibrosis, and dysfunction [56]. Nrf2 played a crucial role in the protective effect of SFN, at least on HG-induced fibrotic response in cultured cardiomyocytes, since SFN lost this effect in the presence of *Nrf2* siRNA [56]. Similarly, in a mouse model of type 2 diabetes (T2DM), SFN was able to activate Nrf2 antioxidant signaling, the effect of which restored the oxidative stress-induced inhibition of liver kinase B1/5′ AMP-activated protein kinase (LKB1/AMPK) signaling pathway and prevented T2DM-induced lipotoxicity and cardiomyopathy [57]. The crucial role of Nrf2 in mediating the protection by SFN against DCM was further demonstrated by using Nrf2 knockout mice [31]. Moreover, metallothionein, a potent antioxidant [58], was identified to be a downstream target of Nrf2 and predominantly mediated SFN's protective effects on diabetic nephropathy [18] and DCM [31]. The effect of SFN has been tested in a double-blind clinical trial of T2DM, showing a significant improvement of insulin resistance [59]. In addition to the improvement of insulin resistance [59], SFN has the advantage of ameliorating DCM and therefore has a good potential for the use in future clinical trials of DCM.

### 4.2. Curcumin

Curcumin is a natural compound isolated from turmeric and has been widely used in indigenous medicine. Attention has been paid to the antioxidative effect of curcumin on DCM [60]. Curcumin was found to reduce myocardial capillary sclerosis [61]; attenuate cardiac tissue damage, myocardial cell hypertrophy, and apoptosis; reduce extracellular protein accumulation; and preserve left ventricular function [62–64] in the hearts of STZ-induced diabetic rats. Mechanistically, curcumin was found to increase HO-1 [64], catalase (CAT), superoxide dismutase (SOD), and GSH [61]. In addition, the ability to reduce the expression of the NOX subunits p22 phox, p47 phox, p67 phox, and gp91 phox could also account for curcumin's protective effects on DCM [62, 63]. In HG-cultured neonatal rat cardiomyocytes, curcumin suppressed the expression of the NOX subunit Rac1 and reduced HG-induced oxidative stress, the effect of which inhibited the HG-induced apoptotic cell death [65].

Although curcumin was found to have antioxidative effects on DCM, the exact target through which curcumin exerted the functions remained unclear. Gene knockout and silencing approaches could aid the investigation of the exact mechanism of antioxidative function induced by curcumin. Furthermore, curcumin exhibits a poor bioavailability in plasma and in target tissues which may hinder its therapeutic efficacy [22]. Therefore, the improvement of the pharmacokinetics and the increase in the plasma concentration of curcumin are significant therapeutic targets in the application of curcumin for the treatment of DCM. Recently, C66, a curcumin derivative with much a higher bioavailability [22, 66], was found to activate Nrf2 and its downstream antioxidants in the kidneys and aortas of the STZ-induced diabetic mice [22, 67]. Nrf2 played a prominent role in the C66 protection against diabetic nephropathy, since C66 lost partial, but significant, protection against the DM-induced renal damage [22] in the *Nrf2* knockout mice. Future studies need to focus on the bioavailability and off-target effects of curcumin.

### 4.3. Icariin

Icariin was reported to inhibit mitochondrial oxidative stress and increase SOD activity in the hearts of the STZ-induced diabetic rats. Icariin was shown, in this study, to reduce myocardial collagen deposition, inhibit ventricular hypertrophy, reverse the DM-induced body weight loss, and improve cardiac function [68]. These results may indicate the efficiency of inhibiting mitochondrial ROS generation and increasing antioxidant capacity in ameliorating DCM. However, blood glucose levels were not indicated by this report, the result of which might be important to know whether the amelioration of DCM was caused by the icariin reduction of oxidative stress or by the amelioration of DM.

### 4.4. Flos Puerariae (FPE)

FPE was shown to inhibit gp91 phox and p47 phox, the two subunits of NOX in the hearts of STZ-induced C57BL/6J diabetic mice [69]. Additionally, FPE inhibited the DM-induced ROS generation and enhanced the activity of SOD and glutathione peroxidase (GSH-Px), maintained myocardial structure, and attenuated DM-induced apoptotic cardiac cell death [69]. Thus, FPE had the capability to both inhibit NOX expression and upregulate the expression of antioxidants in the hearts of the diabetic mice. Despite the speculation of c-Jun N-terminal kinase and P38 mitogen-activated protein kinase to be the target of FPE, further investigations are needed to elucidate the mechanism of this natural product in the amelioration of DCM.

### 4.5. Betanin

Betanin, extracted from natural pigments, was shown by Han et al. to have a protective effect against high fructose feed-induced diabetic cardiac fibrosis in Sprague-Dawley rats [70]. The DM-induced expression of the cardiac profibrotic factors transforming growth factor *β* (TGF-*β*)1 and connective tissue growth factor were significantly inhibited by betanin. The further mechanistic study revealed the efficacy of betanin in inhibiting the AGE/RAGE signaling, oxidative stress, and NF-*κ*B under the diabetic condition. Given that cardiac fibrosis is a hallmark of DCM and contributes to cardiac dysfunction, the remarkable effect of betanin on the inhibition of fibrotic signaling should attract attention for the future studies on DCM.

### 4.6. Chrysin

Chrysin is a natural flavonoid that has antioxidative activity [71]. In a rat model of myocardial injury induced by STZ followed by isoproterenol injection, chrysin was found to activate PPAR-*γ*; upregulate CAT, MnSOD, and GSH; inhibit AGE/RAGE signaling and oxidative stress; and attenuate apoptosis [71]. These results provided evidence for PPAR-*γ* activation in the potential management of DCM. However, more studies are needed to clarify the exact role of chrysin and other PPAR-*γ* activators in ameliorating DCM.

### 4.7. *Aralia taibaiensis* (sAT)

sAT, with antioxidative property [72, 73], is a traditional Chinese medicine that is frequently used in patients with DM [74]. Recently, sAT was reported to activate Nrf2 signaling and reduce HG and glucose oxidase-induced apoptosis, ROS, and oxidative damage in cardiomyocytes [75]. Nrf2 was the key factor through which sAT exerted the protection, since the effects of sAT were markedly abolished in the presence of the *Nrf2* siRNA.

### 4.8. Magnolia Plant Extracts (BL153 or 4-O-Methylhonokiol)

BL153 showed beneficial effects on high-fat diet-induced cardiac [76] and aortic damage [77], via inhibition of oxidative stress. In addition, it has been shown that 4-O-methylhonokiol (MH), a bioactive constituent of BL153, reduced high-fat diet- (HFD-) induced cardiac pathological changes, including increased heart weight and abnormal echocardiography parameters [78]. The observation of the enhanced Nrf2/HO-1 signaling in the hearts of the MH-treated mice [78] could be responsible for the decreased oxidative stress by MH. Both BL153 and MH had the capability to lower serum lipid level and improve insulin resistance in HFD animal models [79]. The effect of BL153 and MH should be tested in future studies on animal models of T1DM, since Nrf2 was shown to play a key role in protection against DCM in T1DM.

### 4.9. *Abroma augusta* L. Leaf

The effect of *Abroma augusta* L. leaf, a natural product that is traditionally used in treatment of DM in India and South Asia, was tested in a streptozotocin-nicotinamide-induced rat model of T2DM [80]. *Abroma augusta* L. leaf was found to reduce hyperglycemia, hyperlipidemia, membrane disintegration, oxidative stress, and oxidative stress-induced cell death in the kidneys and hearts of the diabetic rats [80]. Phytochemical screening revealed the presence of taraxerol, flavonoids, and phenolic compounds in *Abroma augusta* L. leaf [80]. Therefore, the specificity and off-target effects of this natural extract needs further investigation, although the doses provided in this study did not produce side effects in the diabetic rats.

### 4.10. *Aegle marmelos* Leaf Extract (AME)

AME was studied in a rat model of alloxan-induced DM for its effect on early-stage DCM [81]. The results showed that AME evidently increased the antioxidants GSH, CAT, and SOD and rescued the DM-induced cardiac necrosis and inflammation [81].

### 4.11. *Dendrobium officinale* Extract (DOE)

DOE, a traditional Chinese medicine, was shown to elevate the antioxidant SOD and decrease the production of malondialdehyde (MDA), the accumulation of lipid, and the expression of the fibrotic factors TGF-*β*, collagen-1, and fibronectin, as well as the inflammatory factors NF-*κ*B, tumor necrosis factor alpha, and interleukin-1 beta, in the hearts of STZ-induced diabetic mice [82]. DOE at 300 mg/kg also had a significant inhibitory impact on hyperglycemia and cardiac hypertrophy [82]. The effects of DOE on DCM were evident. However, the severe cardiac remodeling, such as hypertrophy and fibrosis, during the 8-week period of DM [82], needs to be further confirmed in future studies.

### 4.12. Fermented Rooibos Extract (FRE)

The protective effect of FRE, from the root of a South African plant containing the antioxidant aspalathin [83, 84], was evaluated on DCM in a STZ-induced rat model of DM [84]. The results showed that FRE preserved GSH activity in the cardiomyocytes isolated from the diabetic rats and prevented the cells from H_2_O_2_ or an ischemic solution-induced generation of ROS and apoptosis [84]. This protective effect was more prominent as compared with that of another antioxidant vitamin E [84].

### 4.13. *Ficus racemosa* Bark Extract

The Indian medicine *Ficus racemosa* bark extract, possessing antioxidant activity, was tested in STZ-induced diabetic rats, for its effect on DCM [85]. The extract was found to enhance the activity of SOD and reduce the level of MDA in the hearts of the diabetic rats [85].

### 4.14. *Ginkgo biloba* Extract

*Ginkgo biloba* has various uses in traditional medicine and as a source of food [86, 87]. Fitzl et al. treated STZ-induced diabetic rats with *Ginkgo biloba* extract and found that *Ginkgo biloba* extract could prevent the loss of myofibrils and the reduction of cardiomyocyte diameter [88]. *Ginkgo biloba* extract was able to increase cardiac SOD activity, without altering the mRNA of inducible nitric oxide synthase [88]. Therefore, the protective effect of this natural product on DCM could be due to its action in scavenging upon DM-induced free radicals, but not blocking the source of ROS.

### 4.15. Kalpaamruthaa

The effect of kalpaamruthaa on DCM was tested in a rat model of T2DM [89]. Kalpaamruthaa was found by Latha et al. to reduce the expression of NOX and endothelial nitric oxide synthase, the effects of which led to a decrease in DM-induced accumulation of cardiac lipid peroxides, proinflammatory cytokines, matrix metalloproteinase-2 and matrix metalloproteinase-9, and cardiac remodeling [89]. A subsequent study by the same group showed that kalpaamruthaa inhibited DM-induced cardiac lipid accumulation, chromatin condensation, and marginalization; increased hepatic antioxidants; improved insulin resistance; and lowered blood glucose level [90]. The ability to inhibit PKC-*β* and enhance Akt activity was suggested to account for the protective effect of kalpaamruthaa on DCM [90]. In a following study, the same group further observed that kalpaamruthaa could increase pancreatic antioxidants and reduce pancreatic lipid peroxides and carbonyl content, markers of injury in the plasma, heart, and liver [91]. The decreased cardiac expression of protease-activated receptor-1 by kalpaamruthaa was indicated for the cardiac protection by kalpaamruthaa [91]. These studies addressed the beneficial role of kalpaamruthaa in protection against DCM. It is needed to clarify the mechanism of this natural product, since it is still unclear whether the protection was due to kalpaamruthaa's alteration of DM or was as a result of other mechanisms. Moreover, several factors were suggested to be the key targets of kalpaamruthaa. It would be helpful to elucidate the exact key factor through which kalpaamruthaa exerts the function by using gene silencing or gene knockout models.

### 4.16. North American Ginseng (NAG)

NAG (*Panax quinquefolius*) is known to have multiple pharmacological functions due to its diverse phytochemical constituents. NAG was studied for its effect on DCM in both STZ-induced type 1 diabetic mice and DB/DB spontaneous type 2 diabetic mice [92]. NAG had the capability to reduce the DM-induced expression of cardiac extracellular matrix proteins and vasoactive factors, as well as cardiac hypertrophy and dysfunction in both types of the mice [92]. The DM-induced cardiac oxidative stress was diminished by NAG, the effect of which might mediate NAG's protection against DCM [92]. Given the diverse phytochemical constituents within NAG, the outcome must be a combination of all the effects exerted by all the constituents. It would be interesting to clarify the key targets of the constituents that may play the major roles in the effect of NAG on DCM. As a result, the most effective constituent and the most potent target could be screened out. This may increase the bioavailability and specificity.

### 4.17. *Pongamia pinnata*

*Pongamia pinnata* is a traditional medicine used in the treatment of DM, and its effect on DCM was tested by using STZ/nicotinamide-induced type 2 diabetic rats [93]. *Pongamia pinnata* decreased blood glucose level, increased the antioxidants SOD and GSH in the hearts of the diabetic mice, and exhibited cardiac protection against DM-induced oxidative damage, biomarkers for cardiac injury, cardiac remodeling, and dysfunction [93]. The exact target of this natural product was not indicated in this study, which needs to be further elucidated in future studies.

### 4.18. *Syzygium cumini*

*Syzygium cumini* was evaluated in H9c2 cells for its protection against glucose stress-induced injury and was found to attenuate high glucose-induced cell hypertrophy and accumulation of extracellular matrix [94], which are hallmarks of long-term diabetic complications [95, 96], including DCM [97].

## 5. Clinical Trials

Despite the reports of animal and cell experiments on the effects of natural products on DCM (Table 1), the use of natural products in clinical trials, to date, has not been conducted. However, the clinical trials of antioxidative natural products in the management of DM should also benefit DCM and may provide clues for the future clinical trials of DCM. Therefore, the natural products used in clinical trials of DM, in terms of their efficacy to inhibit oxidative stress, are listed in Table 2.

### 5.1. *Aloe vera* Inner Leaf Gel Powder

*Aloe vera* inner leaf gel powders (UP780 and AC952) were used in patients with impaired fasting glucose or impaired glucose tolerance during an 8-week period in a double-blind, placebo-controlled study [98]. It was reported that both AC952 and UP780 could markedly reduce fasting glucose and improve glucose tolerance and lipoprotein levels in the plasma [98]. However, the reduction of oxidative stress marker urinary F2-isoprostanes was solely noted for UP780 compared with the placebo.

### 5.2. Black Tea

Mahmoud et al. tested the effects of black tea ingestion on the secretion of inflammatory cytokines and metabolic biomarkers in 30 patients with T2DM [99]. The results indicated that treatment with black tea at 200 or 600 ml per day, for 12 weeks, resulted in significantly decreased glycosylated hemoglobin levels and decreased total serum cholesterol levels and the markers of oxidative stress. Furthermore, the supplementation of black tea could inhibit the inflammatory response including an increase in regulatory T cell secretion and a decrease in the production of proinflammatory cells [99].

### 5.3. Chamomile Tea

A single-blind randomized controlled clinical trial was conducted on 64 patients with T2DM to evaluate the effect of the antioxidative natural product chamomile tea [100]. Chamomile tea, at 3 g/150 ml, 3 times per day, was administered for 8 weeks and was shown to decrease the concentration of serum glycosylated hemoglobin, MDA, insulin, and improved insulin resistance [100]. Additionally, chamomile tea increased total antioxidant capacity, SOD, GSH, and CAT by 6.81%, 26.16%, 36.71%, and 45.06%, respectively [100]. This trial indicates that intake of chamomile tea could benefit glycemic control and antioxidant status in patients with T2DM.

### 5.4. *Nigella sativa*

The long-term effect of *Nigella sativa* on T2DM patients taking standard hypoglycemic drugs was evaluated [101]. The 1-year treatment with *Nigella sativa* led to elevated serum total antioxidant activity and the levels of GSH and SOD, as compared with the non-*Nigella sativa*-treated group [101]. Additionally, a significant decrease in fasting blood glucose and glycosylated hemoglobin and an improvement in insulin resistance and *β*-cell activity were observed in the *Nigella sativa*-treated group [101]. These findings suggest the potential of *Nigella sativa* supplementation as an alternative method to benefit patients with T2DM.

### 5.5. *Phyllanthus emblica* Extract (PEE)

PEE has a rich source of vitamin C, which is an important antioxidant that prevents platelet aggregation in healthy individuals and patients with coronary artery disease [102]. The 10-day treatment with PEE, at a dose of 500 mg twice per day, was well tolerated in patients with T2DM, exhibited inhibitory effect on platelet aggregation, and prolonged the bleeding and clotting time, the effects of which were similar to those of the daily treatment with 75 mg clopidogrel or ecosprin [102].

### 5.6. Aged Garlic Extract

Aged garlic extract has antioxidative and antihyperglycemic effects [103–105]. Supplementation of aged garlic extract in the diet reduced oxidative stress and improved endothelial dysfunction in humans [106, 107]. In a double-blind randomized placebo-controlled crossover pilot trial in patients with T2DM and high cardiovascular risk (30% risk of a cardiovascular event in the next 10 years), the 4-week treatment with aged garlic extract, administered daily at 1200 mg, did not produce significant beneficial effects on body weight, blood pressure, lipids, insulin resistance, and biomarkers of endothelial dysfunction, oxidative stress, and inflammation [108]. Although the authors indicated that the recruitment of patients with higher cardiovascular risk or the supplementation of aged garlic extract for a longer period would produce more pronounced effects [108], the mechanism, dose, and safety of this natural product should be further explored in future studies.

### 5.7. *Salvia miltiorrhiza* Hydrophilic Extract (SMHE)

A randomized controlled clinical study was conducted, using SMHE, to test the protective and antioxidative properties of the extract in diabetic patients with chronic heart disease [109]. SMHE was administrated at a dose of 5 g, twice per day, to the patients for 60 days in addition to their antihyperglycemic therapies. The serum MDA level was decreased by SMHE on day 30. On day 60, the serum GSH, SOD, paraoxonase, and glutathione reductase were significantly increased by SMHE. The lipid levels were not altered by SMHE [109]. This study indicates that SMHE has antioxidative activity and attenuates oxidative stress in diabetic patients with chronic heart disease. Determination of serum biomarkers for cardiac injury, cardiac remodeling, and cardiac dysfunction would be helpful in order to know the cardiac protective effect of SMHE under the diabetic condition.

## 6. Conclusions

Natural products with antioxidative properties have been shown to ameliorate DCM in animal models and cardiomyocytes. Although no natural product has been used in clinical trials specifically targeting DCM, several have been used in clinical trials in patients with DM, even as traditional medicines for the treatment of DM for many years. The capability of the natural products in enhancing serum antioxidative activity and reducing serum oxidative stress may also be beneficial for the amelioration of DCM. Given the antioxidative and even blood glucose-lowering effects of the natural products, they have a great potential for the future clinical use as alternative medicines for the management of DM and DCM.

Despite the bright future of the natural products in the treatment of DCM, a few challenges should be carefully considered before the use of natural products in clinical trials. Natural products have multiple targets. This fact may lead to off-target effects, including both the beneficial and the detrimental. Hence, more selective compounds should be developed. The development of SFN and curcumin, isolated from natural products, is a good example, although the two compounds have multiple targets. In-depth mechanisms need to be elucidated, the result of which can provide a solid basis for the development of novel high-selective compounds. In addition, the blood glucose-lowering effect of some natural products is beneficial. However, whether or not natural products lead to unstable blood glucose level in diabetic patients is a concern. Thus, attention should be paid to the fine control of blood glucose level using the combination of standard medicines and natural products as alternative medicines in the treatment of DM or DCM.

In summary, natural products with antioxidative profiles in the management of DCM on one hand have great potentials and face great challenges on the other. The success of natural products in DCM requires extensive studies on the mechanism, specificity, bioavailability, and drug-to-drug interactions.

## Figures and Tables

**Table 1:** Effects of natural products on diabetic cardiomyopathy.

Name	Model	Dose	Target	Effect	Ref.
Sulforaphane	STZ-induced diabetic C57BL/6J mice	0.5 mg/kg/d, for 3 months	Nrf2	Cardiac oxidative damage ↓, inflammation ↓, hypertrophy ↓, fibrosis ↓, and dysfunction ↓	[56]
	HFD/STZ-induced diabetic C57BL/6J mice	0.5 mg/kg/d, for 4 months	Nrf2	Cardiac LKB1/AMPK pathway ↑, lipotoxicity ↓, fibrosis ↓, inflammation ↓, and dysfunction ↓	[57]
	HFD/STZ-induced diabetic C57BL/6J WT and *Nrf2* KO mice and 129 s WT and *Mt* KO mice	0.5 mg/kg/d, for 4 months	Nrf2	Cardiac MT ↑, HO-1 ↑, NQO1 ↑, oxidative damage ↓, inflammation ↓, fibrosis ↓, hypertrophy ↓, and dysfunction ↓	[31]

Curcumin	STZ-induced diabetic Wistar rats	200 mg/kg/d, for 6 weeks	Free radicals	Myocardial capillary sclerosis ↓	[61]
	STZ-induced diabetic Wistar rats	100 or 200 mg/kg/d, for 16 weeks	AGEs/RAGE, NOX subunits, and SOD	Myocardial dysfunction ↓, cardiac fibrosis ↓, AGE accumulation ↓, oxidative stress ↓, inflammation ↓, apoptosis ↓, phosphorylation of Akt and GSK-3*β* ↑	[62]
	High glucose-treated neonatal rat cardiomyocytes	10 μmol/L, for 30 min	NOX subunits	HG-induced oxidative stress and apoptosis ↓	[65]
	STZ-induced diabetic Sprague-Dawley rats	100 mg/kg/d, for 8 weeks	PKC, NOX subunits, and TGF-*β*	Blood glucose ↓, cardiac oxidative stress ↓, lipid peroxidation ↓, antioxidant activity ↑, cardiomyocyte hypertrophy ↓, myocardial fibrosis ↓, left ventricular dysfunction ↓	[63]
	STZ-induced diabetic rats	20 mg/kg/d, for 45 days	HO-1 ↑	Expression of ANP, MEF2A, MEF2C, and P300 ↓, left ventricular function ↑	[64]

Icariin	STZ-induced diabetic Sprague-Dawley rats	30 or 120 ml/kg/d, for 8 weeks	Mitochondrial ROS	Myocardial collagen deposition ↓, ventricular hypertrophy ↓, body weight loss ↓, cardiac function ↑	[68]

Flos Puerariae	STZ-induced diabetic C57BL/6J mice	100 or 200 mg/kg/d, for 10 weeks	Expression of NOX and the antioxidants SOD and GSH	Cardiac remodeling↓, apoptotic cardiac cell death ↓	[69]

Betanin	High fructose feed-induced diabetic Sprague-Dawley rats	25 or 100 mg/kg/d, for 60 days	AGEs/RAGE, oxidative stress, and NF-*κ*B	Cardiac fibrosis ↓, TGF-*β*1 and CTGF ↓	[70]

Chrysin	STZ-induced diabetic Wistar rats	60 mg/kg, for 28 days	PPAR-*γ*	Cardiac CAT ↑, MnSOD ↑, GSH ↑, AGEs/RAGE ↓, oxidative stress ↓, apoptosis ↓, cardiac dysfunction ↓	[71]

*Aralia taibaiensis*	High glucose-treated H9c2 cells	25, 50, or 75 *μ*g/ml	Nrf2	Apoptosis ↓, ROS ↓, and oxidative damage ↓	[75]

Magnolia plant extract	High-fat diet-induced obese C57BL/6 mice	BL153 at 5 or 10 mg/kg/d, for 24 weeks	Not indicated	Cardiac lipid accumulation ↓, inflammation ↓, oxidative stress ↓, and apoptosis ↓.	[76]
	High-fat diet-induced obese C57BL/6 mice	4-O-methylhonokiol at 0.5 or 1.0 mg/kg/d, for 24 weeks	Nrf2/HO-1, Akt2	Cardiac oxidative stress ↓, lipid accumulation ↓, hypertrophy ↓, and dysfunction ↓	[78]

*Abroma augusta* L. leaf	STZ/nicotinamide-induced type 2 diabetic rats	100 or 200 mg/kg/d, for 4 weeks	Not indicated	Hyperglycemia ↓, hyperlipidemia ↓, membrane disintegration ↓, cardiac oxidative stress and oxidative stress-induced cell death ↓	[80]
*Aegle marmelos* leaf extract	Alloxan-induced diabetic rats	200 mg/kg/d, for 14 days	GSH, CAT, and SOD	Cardiac necrosis ↓ and inflammation ↓	[81]

*Dendrobium officinale* extract	STZ-induced Kunming diabetic mice	300 mg/kg/d, for 8 weeks	SOD	Cardiac MDA ↓, lipid accumulation ↓, and the expression of inflammatory and fibrotic factors ↓	[82]

Fermented rooibos extract	H_2_O_2_-treated cardiomyocytes isolated from the hearts of STZ-induced rats	1 or 10 *μ*g/ml, for 6 hours	GSH	ROS generation ↓, apoptosis ↓	[84]

*Ficus racemosa* stem bark extract	STZ-induced diabetic Wistar rats	200 or 400 mg/kg/d, for 8 weeks	SOD	Cardiac MDA ↓	[85]

*Ginkgo biloba*	STZ-induced diabetic rats	100 mg/kg/d, for 3 months	SOD	Creatine kinase activity ↑, myofibril loss ↓, reduction of myocyte diameter ↓	[88]

Kalpaamruthaa	HFD/STZ-induced diabetic Sprague-Dawley rats	200 mg/kg/d, for 28 days	NOX, eNOS	Cardiac lipid peroxides ↓, proinflammatory cytokines ↓, matrix metalloproteinase-2 and matrix metalloproteinase-9 ↓, cardiac remodeling ↓	[89]
	HFD/STZ-induced diabetic Sprague-Dawley rats	200 mg/kg/d, for 28 days	PKC-*β*/Akt	Cardiac lipid accumulation ↓, chromatin condensation and marginalization ↓, hepatic antioxidants ↑, insulin resistance ↓, blood glucose ↓	[90]
	HFD/STZ-induced diabetic Sprague-Dawley rats	200 mg/kg/d, for 28 days	Cardiac expression of protease-activated receptor-1	Pancreatic antioxidants ↑, pancreatic lipid peroxides and carbonyl content ↓, markers of injury in the plasma, heart, and liver↓	[91]

North American ginseng	STZ-induced diabetic C57BL/6J type 1 diabetic mice or db/db type 2 diabetic mice	200 mg/kg/d, for 2 or 4 months	Oxidative stress	Cardiac extracellular matrix proteins and vasoactive factors ↓, hypertrophy ↓, dysfunction ↓	[92]

*Pongamia pinnata*	STZ/nicotinamide-induced type 2 diabetic rats	100 mg/kg/d, for 4 months	Not indicated	Cardiac SOD ↑, GSH ↑, MDA ↓, remodeling, dysfunction ↓, biomarkers for cardiac injury ↓, blood glucose ↓	[93]

*Syzygium cumini*	High glucose-treated H9c2 cells	9 *μ*g/ml	ROS	Hypertrophy ↓, accumulation of extracellular matrix ↓	[94]

AGEs: advanced glycosylation end products; AMPK: 5′ AMP-activated protein kinase; ANP: atrial natriuretic peptide; CAT: catalase; CTGF: connective tissue growth factor; eNOS: endothelial nitric oxide synthase; GSH: glutathione; GSK-3*β:* glycogen synthase kinase 3 beta; HFD: high-fat diet; HG: high glucose; HO-1: heme oxygenase-1; KO: knockout; LKB1: liver kinase B1; MDA: malondialdehyde; MEF2A: myocyte-specific enhancer factor 2A; MEF2C: myocyte-specific enhancer factor 2C; MT: metallothionein; NF-*κ*B: nuclear factor kappa-light-chain-enhancer of activated B cells; NOX: NADPH oxidase; NQO1: NADPH quinone oxidoreductase; Nrf2: nuclear factor erythroid 2-related factor 2; PKC-*β*: protein kinase C-beta; PPAR-*γ*: peroxisome proliferator-activated receptor-gamma; RAGE: receptor for AGEs; ROS: reactive oxygen species; SOD: superoxide dismutase; STZ: streptozotocin; WT: wild type; ↑: activation or improvement; ↓: inhibition or downregulation.

**Table 2:** Antioxidative natural products in clinical trials of diabetes.

Name	Disease	Dose	Effect	Ref.
*Aloe vera* inner leaf gel powder	Patients with impaired fasting glucose or glucose tolerance	UP780 or AC952 at 500 mg, twice a day, for 8 weeks	Fasting glucose ↓, glucose tolerance ↑, serum lipoprotein levels ↓ (both UP780 and AC952), urinary F2-isoprostanes ↓ (UP780)	[98]
Black tea	T2DM	2.5 g/200 ml or 7.5 g/600 ml/d, for 12 weeks	Serum glycosylated hemoglobin ↓, cholesterol ↓, markers of oxidative stress ↓, regulatory T cell secretion ↑, proinflammatory cells ↓	[99]
Chamomile tea	T2DM	3 g/150 ml, 3 times a day, for 8 weeks	Serum glycosylated hemoglobin ↓, malondialdehyde ↓, insulin ↓, insulin resistance ↓, total antioxidant capacity ↑, SOD ↑, GSH ↑ and CAT activity ↑	[100]
*Nigella sativa*	T2DM	2 g/day, for 1 year	Fasting blood glucose ↓, glycosylated hemoglobin ↓, glucose homeostasis ↑, total antioxidant capacity ↑, the levels of GSH ↑ and SOD ↑	[101]
*Phyllanthus emblica*	T2DM	500 mg, twice daily, for 10 days	Platelet aggregation ↓, bleeding and clotting time ↑	[102]
Aged garlic extract	Patients with T2DM and high cardiovascular risk	1200 mg/d, for 4 weeks	No significant beneficial effects on body weight, blood pressure, lipids, insulin resistance, and biomarkers of endothelial dysfunction, oxidative stress, and inflammation.	[108]
*Salvia miltiorrhiza* hydrophilic extract	Diabetic patients with chronic heart disease	5 g, twice per day, for 60 days	Serum MDA ↓, GSH ↑, SOD ↑, paraoxonase ↑, and glutathione reductase ↑	[109]

T1DM: type 1 diabetes; T2DM: type 2 diabetes; ↑: activation or improvement; ↓: inhibition or downregulation. Other abbreviations are the same as in Table 1.
